# The Potential Role of Cycloastragenol in Promoting Diabetic Wound Repair In Vitro

**DOI:** 10.1155/2019/7023950

**Published:** 2019-12-18

**Authors:** Yi Cao, Li Xu, Xiaohong Yang, Yuan Dong, Hongbin Luo, Fengling Xing, Qiongxiang Ge

**Affiliations:** ^1^Department of Dermatology, The First Affiliated Hospital of Zhejiang Chinese Medicine University, Hangzhou, Zhejiang 310006, China; ^2^Department of Anorectal Surgery, The First Affiliated Hospital of Zhejiang Chinese Medicine University, Hangzhou, Zhejiang 310006, China; ^3^Department of Pathology, The First Affiliated Hospital of Zhejiang Chinese Medicine University, Hangzhou, Zhejiang 310006, China; ^4^Zhejiang Chinese Medicine University, Hangzhou, Zhejiang 310015, China

## Abstract

**Background:**

Refractory wound healing is a severe complication of diabetes with a significant socioeconomic burden. Whereas current therapies are insufficient to accelerate repair, stem cell-based therapy is increasingly recognized as an alternative that improves healing outcomes. The aim of the present study is to explore the role of cycloastragenol (CAG), a naturally occurring compound in *Astragali Radix*, in ameliorating refractory cutaneous wound healing in vitro, which may provide a new insight into therapeutic strategy for diabetic wounds.

**Methods:**

Human epidermal stem cells (EpSCs) obtained from nine patients were exposed to CAG, with or without DKK1 (a Wnt signaling inhibitor). A lentiviral short hairpin RNA (shRNA) system was used to establish the telomerase reverse transcriptase (TERT) and *β*-catenin knockdown cell line. Cell counting kit-8, scratch wound healing, and transwell migration assay were used to determine the effects of CAG in cell growth and migration. The activation of TERT, *β*-catenin, and c-Myc was determined using real-time qPCR and western blot analysis. Chromatin immunoprecipitation (ChIP) was performed to evaluate the associations among CAG, TERT, and Wnt/*β*-catenin signals.

**Results:**

CAG not only promoted the proliferation and migration ability of EpSCs but also increased the expression levels of TERT, *β*-catenin, c-Myc. These effects of CAG were most pronounced at a dose of 0.3 *μ*M. Notably, the CAG-promoted proliferative and migratory abilities of EpSCs were abrogated in TERT and *β*-catenin-silenced cells. In addition, the ChIP results strongly suggested that CAG-modulated TERT was closely associated with the activation of Wnt/*β*-catenin signaling.

**Conclusion:**

Our data indicate that CAG is a TERT activator of EpSCs and is associated with their proliferation and migration, a role it may play through the activation of Wnt/*β*-catenin signaling.

## 1. Introduction

In recent years, wound repair, especially for chronic nonhealing wounds, has emerged as a global public health issue that concerns physical and mental health in both type 1 and type 2 diabetes [1]. Nonhealing wounds are a devastating complication that precedes most diabetes-related amputations and requires over US$20 billion of treatment in the USA annually [2]. In China, 25% of diabetic patients suffer from a diabetic foot ulcer, which typically costs around $50,000 annually to treat because of its refractory nature and the need for continuous care. Enormous effort has therefore been invested in developing innovative and efficient therapies for diabetic nonhealing wounds [3].

Wound healing is a complex process that requires an inflammatory reaction, wound clotting, re-epithelialization, remodeling regulation, and control of stem cells [4, 5]. The epidermal stem cell (EpSC), as a specific stem cell of skin tissue, is increasingly recognized as a crucial component in diabetic wound healing. Previous research has demonstrated that, compared with exogenous EpSCs, resident EpSCs are more important in tissue remodeling and wound closure; the loss of resident EpSCs, impairment of their migration capacity, and/or excessive differentiation may lead to delay in wound healing. Accordingly, stem cell-based therapy is gaining recognition as a way to improve healing outcomes [6].

In terms of mechanism, there is an accumulation of evidence that telomere is critically important for wound repair and that the dysfunction of telomere has a negative effect on wound healing by impairing the ability of EpSCs to proliferate or migrate [7]. The Wingless/integrase-1 (Wnt) pathway is an evolutionarily conserved set of signals that plays a critical role in embryo development and tissue homeostasis across species [8]. There is clear evidence in mammals that it can be reactivated in response to injury and that it is closely related to cell proliferation, differentiation, and migration during tissue regeneration [9, 10]. Researchers have suggested that the Wnt/beta-catenin pathway is involved in inflammatory response to diabetic ulcers, wound proliferation, wound remodeling, and stem cells [4]. In a prospective, randomized, controlled animal study, Yang and colleagues found that a lack of telomerase in mice was invariably associated with inhibited activation of Wnt/*β*-catenin signaling, which could be improved with the administration of Wnt activator [11]. The data from their study suggest a potential relationship between telomerase and Wnt/*β*-catenin signaling. However, despite this progress, current therapies and research into the relevant mechanisms remain inadequate.

Cycloastragenol (CAG), a triterpenoid saponin compound and a hydrolysis product of *Astragali Radix*, has been confirmed to have a variety of pharmacological functions, including antiaging, anti-inflammation, antifibrosis, and protection of the liver and endothelium [12, 13]. Research has indicated that CAG can effectively stimulate telomerase activity and cell proliferation in human neonatal keratinocytes, which suggests that it is a potent telomerase activator for neuronal repair [14]. However, to our knowledge, CAG has not yet been studied in the context of diabetic wound healing. Our primary aim was therefore to explore the role of CAG in regulating cell proliferation, migration, telomerase reverse transcriptase (TERT) expression, and activation of the Wnt/*β*-catenin pathway in EpSCs, in order to provide new insight into therapeutic strategy for diabetic wounds.

## 2. Materials and Methods

### 2.1. Isolation, Culture, and Identification of Human EpSCs

Human foreskin samples were obtained from seven healthy donors. Primary EpSCs were isolated using a standard protocol adopted from Yang et al. [15], which allowed the retention of many characteristics of EpSCs, including morphology and antigens. Flow cytometry and immunofluorescence (IF) staining with antibodies of K19 (1 : 200 diluted, ab52625, Abcam, UK), K15 (1 : 100 diluted, ab80522, Abcam), K14 (1 : 200 diluted, ab9220, Abcam), CD34 (1 : 200 diluted, ab81289, Abcam), and *β*1-integrin (1 : 250 diluted, ab134179, Abcam) were, respectively, performed to identify the phenotype of our EpSCs. Finally, isolated cells were cultured in a Keratinocyte medium (KM, 2101, cell science) supplemented with 100 U/ml penicillin and 100 *μ*g/ml streptomycin, in a humidified atmosphere of 95% air and 5% CO_2_ at 37°C. All the participants wrote the informed consent, and the experimental procedures were approved by the Ethical Review Board at the First Affiliated Hospital of Zhejiang Chinese Medicine University (No. 2017-KL-024-01).

### 2.2. Plasmid Preparation, RNA Interference, and Stable Transfection

Plasmid constructs of TERT and *β*-catenin were manufactured by GenScript (Piscataway, USA). The sequence of short-hairpin RNA (shRNA) for target genes was provided by the authors. The plasmids were constructed using vector pRNAi-U6.2 (+) as the backbone and Hpa I and Xho I as the cloning site. The primer of shRNAs were synthesized by Genepharma Corp. (Shanghai, China), and the sequences used were as follows: shTERT #1, 5′-GCTGCTTTATTCTCCCATTGA-3′ and 5′- TCGACCTGCTGGAATCTCGTG-3′; shTERT ##2, 5′-GGTTAATAA-GGCTGCAGTTAT-3′ and 5′- TCGACCTGCTGGAATCTCGTG-3′; shTERT ###3, 5′-GCTTATGGCAACCAAGAAAGC-3′ and 5′-TCGACCTGCTGGAATCTCGTG-3′; sh*β*-catenin #1, 5′-GCTCGTGGAGACCATCTTTCT-3′ and 5′-TCGACCTGCTGGAATCTCGTG-3′; sh*β*-catenin ##2, 5′-GGAAGACAGTGGTGAACTTCC-3′ and 5′-TCGACCT-GCTGGAATCTCGTG-3′; sh*β*-catenin ###3, 5′-GGAAGAGTGTCTGGAGCAAGT-3′ and 5′-TCGACCTGCTGGAATCTCGTG-3′. For stable transfection, T293 cells were infected with TERT and *β*-catenin shRNA lentiviral particles and control lentiviruses, according to the manufacturer's instructions (GenePharma, Shanghai, China) and using Lipofectamine® 3000 (Invitrogen, USA). The transfection efficiencies were detected using qRT-PCR and western blot, respectively.

### 2.3. Cell Counting Kit (CCK-8) Assay

Cells (2 × 10^3^ cells per well; three replicates per group) were seeded into a 96-well plate and incubated at 37°C. At the indicated time (0, 1, 2, 3 day or 5 day, and 7 day), the absorbance of cells cultured in different concentrations (0, 0.03, 0.3, 1, and 10 *μ*M) of GCA was measured at 450 nm with 10 *μ*L of CCK-8 reagent (Dojindo, Japan) for approximately 1.5 h. The assay was conducted in six replicate wells for each sample.

### 2.4. Transwell Migration Assay

The cells were distributed on the transwell inserts (Millipore, USA) at a density of 1 × 10^5^ and incubated with GCA (0.3 *μ*M) or with an equal volume of dimethyl sulfoxide (DMSO) as a control for a further period of 24 h. Migrated cells were determined using crystal violet staining. Images of the crystal violet stain were captured using a light microscope (Olympus, Japan). The assay was conducted on three replicates per group, and three parallel experiments were performed.

### 2.5. Scratch Wound Healing Assay

Cells were seeded in six-well plates at 5 × 10^5^ cells per well. After the cells had attached, the monolayer was scratched with a P1000 pipette tip and washed three times with PBS to remove the floating cells. The cells were then exposed to GCA from different groups or an equal volume of DMSO. EpSCs were photographed at 0 h and 24 h after wounding, and the distance between the scratches was taken to represent the proliferative ability of EpSCs in wound healing.

### 2.6. Quantitative Real-Time Polymerase Chain Reaction (qRT-PCR)

Total RNAs of fresh cells were extraction by using the TRIzol reagent (15,596,026, Invitrogen) and then synthesized to complementary DNA according to the instruction of manufacturer (K1691, RevertAid RT Reverse Transcription Kit, Thermo, USA). Real-time PCR reaction (18887320, Roche, Switzerland) was conducted in a Bio-Rad system for 40 cycles, with each cycle consisting of denaturation at 95°C for 5 s and annealing at 60°C for 60 s; the melt curve was 95°C for 60 s and 65°C for 60 s. GAPDH was used as an endogenous control for normalization, and the relative quantitation of target gene expression was quantified using the 2^−ΔΔCT^ method [16]. Primer sequences are listed as follows: TERT forward: 5′-CCGATTGTGAACATGGACTACG-3′ and reverse: 5′-CACGCTGAACAGTGCCTTC-3′; *β*-catenin forward: 5′-CATCTACACAGTTTGATGCTGCT-3′ and reverse: 5′-GCAGTTTTGTCAGTTCAGGGA-3′; c-Myc forward: 5′-GGCTCCTGGCAAAAGGTCA-3′ and reverse: 5′-CTGCGTAGTTGTGCTGATGT-3′; GAPDH forward: 5′-GGAGCGAGATCCCTCCAAAAT-3′ and reverse: 5′- GGCTGTTGTCATACTTCTCATGG-3′.

### 2.7. Western Blot

Western blot was performed as described elsewhere. Briefly, the fresh cells were harvested, washed with PBS, and lysed in lysis buffer. After being boiled, the proteins were resolved on 10% sodium dodecyl sulfate-polyacrylamide gel electrophoresis (SDS-PAGE) gels and then electrotransferred onto polyvinylidene fluoride (PVDF) membranes. Primary antibodies used were as follows: anti-TERT (1 : 1000 diluted, ab32020, Abcam), *β*-catenin (1 : 1000 diluted, 8480, Cell Signaling Technology, USA), c-Myc (1 : 1000 diluted, ab32072, Abcam), and GAPDH (1 : 1000 diluted; 5174, CST). Next day, proteins were incubated with horseradish peroxidase-conjugated secondary antibody and visualized by using the enhanced chemiluminescence method. The intensity of the bands was quantified using the image lab 6.0 system (Bio-Rad Laboratories, USA), and the data were normalized to the GAPDH as loading controls. All western immunoblot analyses were performed three times.

### 2.8. Chromatin Immunoprecipitation (ChIP)

The total protein was extracted and processed according to the Total Protein Extraction Kit for Animal Cultured Cells and Tissue User Manual v.4. Immunoprecipitation was performed with antibodies against TERT (1 : 100 diluted, Abcam) and *β*-catenin (1 : 50 diluted, CST), and a normal rabbit IgG was used as a control. All experiments were performed in triplicate and repeated a minimum of three times.

### 2.9. Statistical Analysis

All data were analyzed using SPSS (version 19.0, USA) and presented as mean ± standard deviation (SD). The Kolmogorov–Smirnov method was used to test whether the data were distributed normally. Statistical analysis was performed by the paired two-sample Student's *t* test, and *P* value <0.05 was considered as statistically significant.

## 3. Results

### 3.1. Identification of Human Primary EpSCs

**Figure 1:**
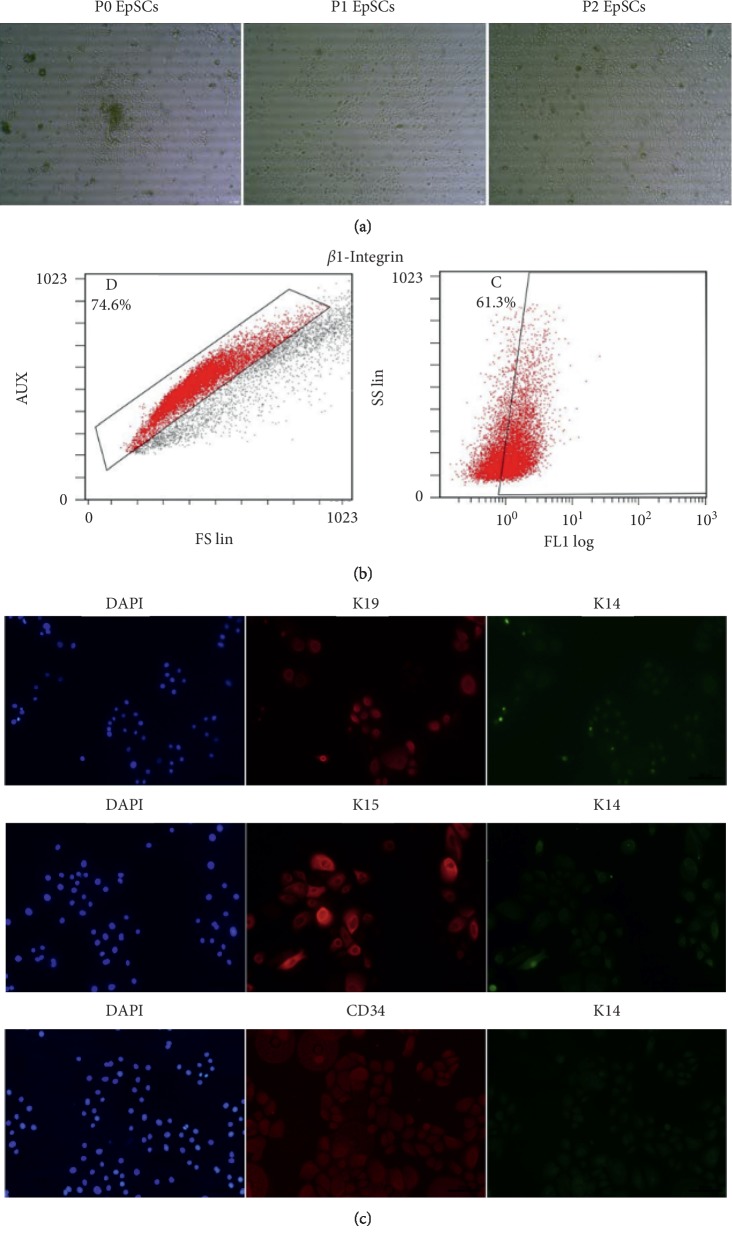
Identification of human primary EpSCs. (a) Cell morphology for primary human EpSCs that of the first, second, and third generations. Original magnification, ×100. (b) Representative flow cytometry that gating to identify *β*1-integrin^+^ cells. (c) Immunofluorescence of cells for K19, K15, CD34, and K14, respectively. Original magnification, ×640.

The isolated primary EpSCs exhibited a normal epithelial-like appearance with close cell-to-cell contacts, which was consistent with other descriptions (Figure 1(a)). To further identify the phenotypes of EpSCs, the expression levels of *β*1-integrin were examined using flow cytometry, and K19, K15, CD34, and K14 were detected using IF staining [15, 17]. As shown, significantly higher levels of *β*1-integrin and K19, K15, and CD34 were detected in our EpSCs. In contrast, there was almost no expression of K14 in these cells, which suggests that successful human primary EpSCs were achieved in our culture conditions (Figures 1(b) and 1(c)).

### 3.2. Proliferation and Migration Ability of Cells

**Figure 2:**
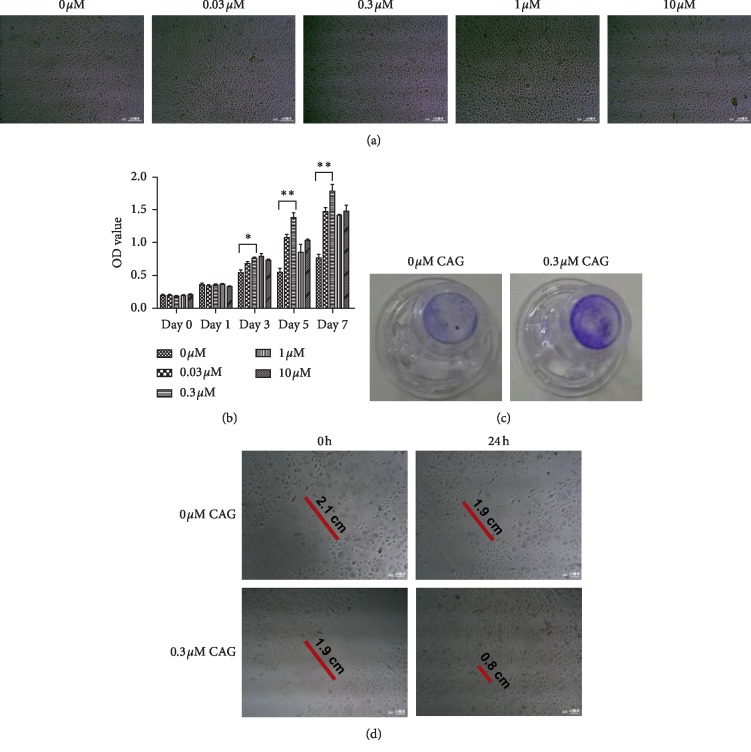
Cell proliferation and migration tests. (a) Representative images of EpSCs morphologic alterations that co-cultured with different concentrations of CAG (0, 0.03, 0.3, 1, and 10 *μ*M). Original magnification, ×100. (b) The cell proliferation ability mediated by different concentrations of CAG up to 7 days. Data presented as mean value ± SD of three independent experiments. Significance level was labeled as follows: ^*∗*^*P* < 0.05, ^*∗∗*^*P* < 0.01, and ^*∗∗∗*^*P* < 0.001. (c) Representative images of Transwell migration assay. (d) Representative images of scratch wound healing assay. Original magnification, ×250.

EpSCs were incubated in the absence or presence of CAG.  Figure 2(a) illustrates the cell morphology of the EpSCs that were cultured with different concentrations of CAG (0, 0.03, 0.3, 1, and 10 *μ*M). Cells in each group clearly displayed a similar, normal epithelial-like appearance, with close cell-to-cell contacts, up to 72 h. Neither low nor high concentrations of CAG were associated with any morphologic alterations in EpSCs under a phase-contrast microscope. Our CCK-8 assay demonstrated that there were no concentration- or time-dependent cytotoxic effects of CAG on these human primary EpSCs over the indicated time spans (0, 1, 3, 5, and 7 days). More importantly, the cell viability of EpSCs increased significantly in co-culture with CAG at days 3, 5, and 7 compared with normal cells, and the changes were especially pronounced at a concentration of 0.3 *μ*M. In other settings, there were no detectable differences in the cell viability of each group (Figure 2(b)). Cell migratory ability was observed using both transwell migration assay (Figure 2(c)) and scratch wound healing assay (Figure 2(d)). These observations showed that CAG effectively promoted the migratory ability of EpSCs at a dose of 0.3 *μ*M up to 24 h. These data strongly support the hypothesis that CAG can play an important role in promoting the proliferation and migration of human primary EpSCs.

### 3.3. Effect of CAG on Telomerase Reverse Transcriptase in Human Primary EpSCs

**Figure 3:**
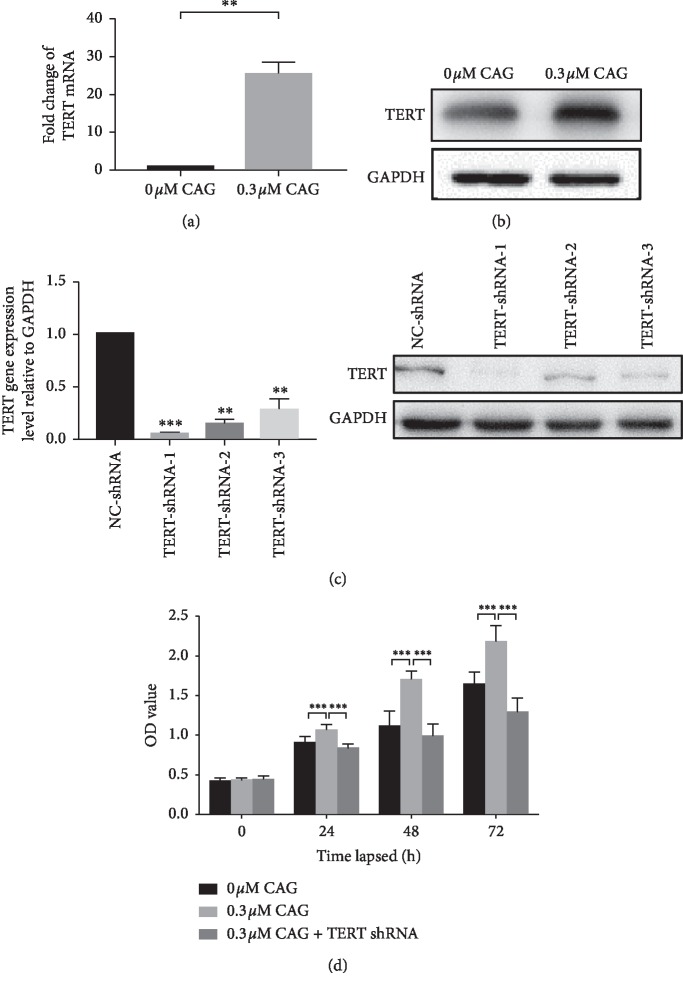
The expression of TERT in primary human EpSCs. (a) Real-time qPCR for TERT. (b) Western blot for TERT. The relative expression levels were normalized to GAPDH. (c) The identification of TERT shRNA. (d) The cell proliferation ability mediated by CAG up to 3 days. All data were presented as the mean ± SD of three independent experiments with triplicate wells. Significance level was labeled as follows: ^*∗*^*P* < 0.05, ^*∗∗*^*P* < 0.01, and ^*∗∗∗*^*P* < 0.001.

Telomerase reverse transcriptase (TERT) is a catalytic component of human telomerase, undetectable in normal somatic cells but upregulated in cancers and stem cells, where telomere length is maintained by telomerase [18]. In the in vitro experiment, we found that the expression of TERT was considerably raised in EpSCs subjected to 0.3 *μ*M of CAG EpSCs (Figure 3(a)). Most importantly, we obtained further confirmation of the role of CAG in TERT-silenced EpSCs (Figure 3(b)). As shown, we found that the viability of EpSCs co-cultured with 0.3 *μ*M CAG was significantly increased compared with their viability in the absence of CAG over the indicated time spans (0, 1, and 3 days). However, in comparisons between CAG-treated and CAG-treated TERT-silenced EpSCs, the latter group persistently showed a detectable decrease in cell viability compared with the former group (Figure 3(c)). Collectively, our findings suggest that CAG can improve the cell proliferation of primary EpSCs via upregulation of TERT.

### 3.4. Effect of CAG on Wnt/*β*-Catenin Signaling in Human Primary EpSCs

**Figure 4:**
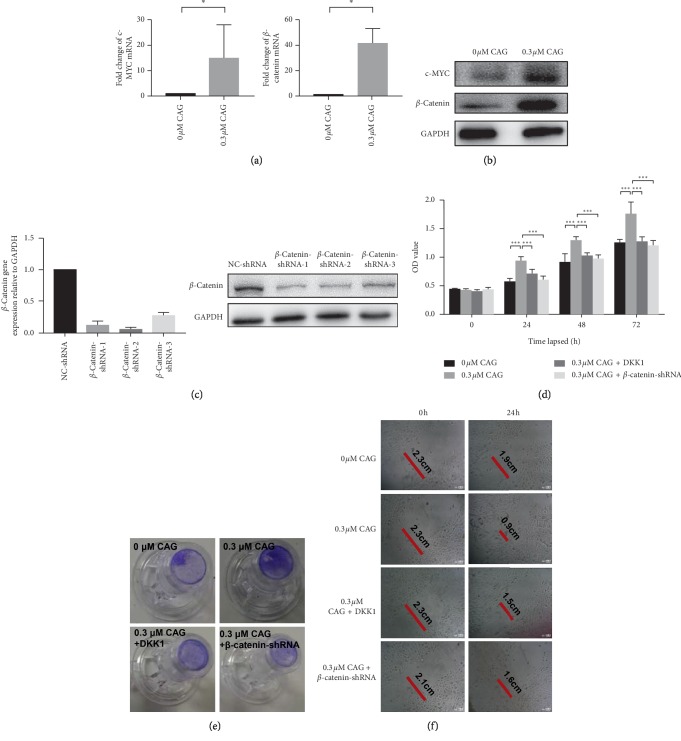
Wnt/*β*-catenin signaling in primary human EpSCs. (a) Real-time qPCR for *β*-catenin and c-Myc. (b) Western blot for *β*-catenin and c-Myc. The relative expression levels were normalized to GAPDH. Comparisons between two groups were determined by the Student's *t* test; significance level was set at ^*∗*^*P* < 0.05, ^*∗∗*^*P* < 0.01, and ^*∗∗∗*^*P* < 0.001. (c) The identification of *β*-catenin shRNA. (d) The cell proliferation ability mediated by CAG up to 3 days. (e) Representative images of Transwell migration assay. (f) Representative images of scratch wound healing assay. Original magnification, ×250.

Since it has been reported that Wnt/*β*-catenin signaling is one of the key regulators for cell proliferation and tissue regeneration [3], we evaluated whether CAG can activate the Wnt/*β*-catenin signaling in EpSCs. As expected, the results of real-time qPCR indicated that the relative expression levels of *β*-catenin and c-Myc were significantly increased in EpSCs treated with CAG compared with those without CAG treatment (Figure 4(a)). Western blot analysis revealed similar alterations of *β*-catenin and c-Myc (Figure 4(b)). Furthermore, we examined the proliferation and migration ability of cells treated with DKK1 (a Wnt signaling inhibitor) and in *β*-catenin-silenced EpSCs (Figures 4(c) and 4(d)). We found that cells administered with DDK and CAG had lower levels of migration ability than those without DDK. Similarly, co-culturing with CAG in *β*-catenin knockout EpSCs resulted in lower migration ability compared with normal cells subjected only to CAG (Figures 4(e) and 4(f)). These data indicate that CAG effectively activates Wnt/*β*-catenin signaling in human primary EpSCs, which is associated with increased cell proliferation and migration ability in EpSCs.

### 3.5. CAG-Improved TERT Expression through Wnt/*β*-Catenin Signaling

**Figure 5:**
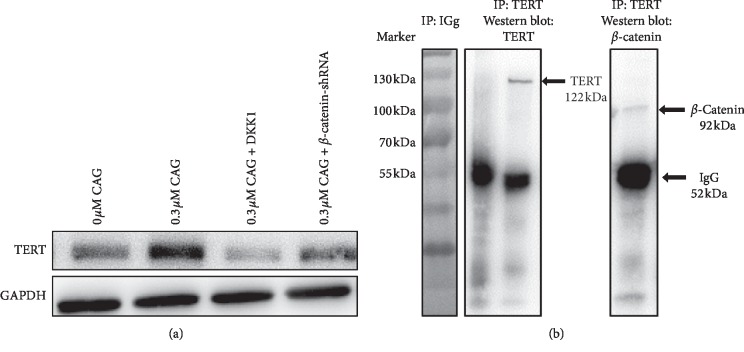
The effect of CAG on both TERT and Wnt/*β*-catenin signaling in human primary EpSCs. (a) Representative blots for TERT in each group. (b) ChIP for TERT, *β*-catenin, and IgG. All data were presented as the mean ± SD of three independent experiments. Significance level was labeled as follows: ^*∗*^*P* < 0.05, ^*∗∗*^*P* < 0.01, and ^*∗∗∗*^*P* < 0.001.

In order to explore the potential mechanism of CAG in diabetic wound healing, we investigated the relationship between TERT and Wnt/*β*-catenin signaling in our EpSCs. As described above, the administration of CAG effectively promoted the expression of TERT. However, the results shown in Figure 5(a) suggest that there was no detectable increase of TERT for EpSCs treated with DDK or subjected to the knockout of *β*-catenin compared with cells without CAG. Interestingly, the ChIP results further demonstrated a close association between TERT and *β*-catenin in EpSCs, which strongly indicates that CAG contributes to the expression of TERT through the activation of Wnt/*β*-catenin signaling (Figure 5(b)).

## 4. Discussion

In this study, we first investigated the role of CAG in the proliferation and migration of EpSCs and the mechanism involved. Our data demonstrated that (1) CAG promotes the proliferation and migration of primary human EpSCs; (2) CAG effectively increases the expression of TERT in primary human EpSCs; (3) there was a significant activation of Wnt/*β*-catenin signaling in the primary human EpSCs after the administration of a certain concentration of CAG; and (4) TERT and Wnt/*β*-catenin signaling was dramatically activated and co-expressed in EpSCs after CAG treatment. These observations suggest that CAG effectively contributes to the proliferation and migration of EpSCs, and this may be related to the increased expression of TERT and the activation of Wnt/*β*-catenin signaling. Our data provide new insight into this mechanism and its potential therapeutic use in experimental diabetic wound repair.

Skin acts as a barrier to foreign pathogens, regulates body temperature, provides sensation, and prevents dehydration of the body [19]. With types of injury in which the skin is torn, cut, or punctured, a well-orchestrated repair process of hemostasis, inflammation, proliferation, and remodeling occurs [2, 20]. Studies show these overlapping but distinct phases involve various inflammatory cells, repair cells and mediators, and cellular responses; the disruption of the cellular and molecular signals in conditions such as diabetes, infection, or radiation exposure may result in inefficient healing [20]. Research has found that loss of resident EpSCs, impairment of migration capacity, and/or excessive differentiation of EpSCs are significantly associated with diabetic wound repair. However, the therapeutic opportunities and the mechanism involved remain to be elucidated.

CAG is an aglycone of astragaloside IV that has various pharmacological actions [21], and it has been shown to be a potent telomerase activator for antiaging, anti-inflammation, endothelial homeostasis, and tissue repair, both in vivo [22] and in vitro [14, 23]. Given the relationships among CAG, telomerase, and the repair process involving hemostasis, inflammation, proliferation, and remodeling, we speculated that CAG may play a protective role in the growth and migration of EpSCs growth, ultimately contributing to wound repair.

First, as expected, we found that CAG promoted the proliferation and migration of primary human EpSCs. Notably, our data also demonstrated that, during the indicated time spans, CAG was not associated with any morphologic alterations in EpSCs, and there were no concentration- or time-dependent cytotoxic effects of CAG on these primary human EpSCs. Most importantly, we identified 0.3 *μ*M as the most effective concentration in terms of contribution to cell proliferation and migration.

Next, we explored whether CAG had a protective role on TERT in primary human EpSCs. TERT, a reverse transcriptase of telomerase has been shown to be involved in certain types of injury through the activation of specific signals, such as NF-kappa B and autophagy [24], and it may have noncanonical functions in regulating the expression of particular genes [18]. As expected, in our real-time qPCR and western blot analysis, we found a significantly increased expression of TERT in EpSCs, which was related to improved proliferation and migration abilities.

Most importantly, we further confirmed that TERT may play this role though Wnt/*β*-catenin signaling. The Wnt/*β*-catenin signaling pathway is known to be crucial for proper organ development. Classically, Wnt signaling has been separated into canonical and noncanonical signaling, with Wnt signaling that relies on the activation of the transcriptional coactivator *β*-catenin designated as canonical [25]. Although it is clear that Wnt/*β*-catenin signaling is of great importance for stem cell growth, maintenance, and turnover of epithelium during homeostasis and in response to injury, there is still some controversy as to the details. For example, Sun et al. proposed that Wnt signaling is a potent regulatory molecule for stem cell turnover and skin regeneration but that it is not well activated in diabetic wounds [26]. Houschyar and colleagues suggested that Wnt signaling is activated by wounding and participates in each subsequent stage of the healing process, from the control of inflammation and programmed cell death to the mobilization of stem cell reservoirs within the wound site [27]. By contrast, few investigators have argued for the protective role of inhibition of Wnt/*β*-catenin pathway in wound repair [28]. Notably, our findings suggest that the activation of Wnt/*β*-catenin contributes to the expression of TERT, eventually promoting cell proliferation and migration. In combination, these results provide useful data for research into the mechanism of diabetic wound healing.

The present study has some limitations. It should be acknowledged that our data propose a general role for CAG in promoting the proliferation and migration of EpSCs, rather than a mechanism involved specifically in the evolution of diabetic wound healing. The in vitro experiment may therefore not be fully representative of the conditions in clinical settings with diabetic patients. Further research using classic animal models or target gene-deficient animals is required to confirm our findings. Furthermore, CAG is an aglycone derivative of astragaloside IV that has various pharmacological actions and regulatory signals [13], and thus the precise mechanism of CAG involved in the treatment of wound repair remains to be explored. Despite these limitations, we are confident that our research provides useful data concerning the potential molecular mechanisms and their therapeutic potential for diabetic wound healing.

In conclusion, our data indicate that CAG can promote the proliferation and migration of EpSCs, and that this is associated with increased expression of TERT in EpSCs. CAG may play this role through activation of Wnt/*β*-catenin signaling.

## Data Availability

The data and materials used and/or analyzed during the present study are available from the corresponding author on reasonable request.

## Abbreviations

EpSCs: Epidermal stem cells
Wnt: Wingless/integrase-1
CAG: Cycloastragenol
TERT: Telomerase reverse transcriptase
IF: Immunofluorescence
shRNA: Short-hairpin RNA
ChIP: Chromatin immunoprecipitation.
